# Privacy and data protection in mobile cloud computing: A systematic mapping study

**DOI:** 10.1371/journal.pone.0234312

**Published:** 2020-06-11

**Authors:** Hussain Mutlaq Alnajrani, Azah Anir Norman, Babiker Hussien Ahmed

**Affiliations:** 1 Faculty of Computing Science and Information Technology, University of Malaya, Kuala Lumpur, Malaysia; 2 Faculty of Computer Science and Information Technology, Albaha University, Albaha, Saudi Arabia; Wuhan University, CHINA

## Abstract

As a result of a shift in the world of technology, the combination of ubiquitous mobile networks and cloud computing produced the mobile cloud computing (MCC) domain. As a consequence of a major concern of cloud users, privacy and data protection are getting substantial attention in the field. Currently, a considerable number of papers have been published on MCC with a growing interest in privacy and data protection. Along with this advance in MCC, however, no specific investigation highlights the results of the existing studies in privacy and data protection. In addition, there are no particular exploration highlights trends and open issues in the domain. Accordingly, the objective of this paper is to highlight the results of existing primary studies published in privacy and data protection in MCC to identify current trends and open issues. In this investigation, a systematic mapping study was conducted with a set of six research questions. A total of 1711 studies published from 2009 to 2019 were obtained. Following a filtering process, a collection of 74 primary studies were selected. As a result, the present data privacy threats, attacks, and solutions were identified. Also, the ongoing trends of data privacy exercise were observed. Moreover, the most utilized measures, research type, and contribution type facets were emphasized. Additionally, the current open research issues in privacy and data protection in MCC were highlighted. Furthermore, the results demonstrate the current state-of-the-art of privacy and data protection in MCC, and the conclusion will help to identify research trends and open issues in MCC for researchers and offer useful information in MCC for practitioners.

## 1. Introduction

In recent years, mobile cloud computing (MCC) is playing a crucial role in connectivity and accessibility to services and applications [1]. MCC is a major area of interest evolving out of mobile devices and cloud computing [1–3]. It is an approach that aims to enable mobile terminals to access robust and reliable cloud-based computing that facilitates the optimal utilization of resources.

As an effect of a major concern of cloud users, the issue of privacy and data protection has received considerable attention in the field. A number of researchers have reported that privacy in the definition adopted by the organization for Economic Cooperation and Development [4] is “any information relating to a recognized or identifiable individual (data subject).” In fact, the concept of privacy has a different perspective, depending on countries, cultures, or jurisdictions.

Recently, researchers have shown an increased interest in MCC. Currently, a considerable number of papers have been published on MCC with a growing interest in privacy and data protection. Along with this advance in MCC, the results of the existing studies in privacy and data protection are not highlighted. Also, no particular research demonstrates the ongoing trends, measures to assess current solutions, and open research issues, including future research directions for privacy and data protection in MCC.

In this study, a systematic mapping study (SMS) was conducted to analyses the existing research literature that addresses privacy and data protection in MCC [3]. In fact, SMS is a clear and precise method of identifying, evaluating, and explaining all obtainable research relevant to a specific research question, thematic area, or phenomenon of importance [3]. Furthermore, the purpose of SMS is to present an adjustable, impartial, and reliable assessment of a particular research topic [3].

The study presented in this paper aims to highlight the results of existing primary studies published in privacy and data protection in MCC to identify current trends and open issues in the domain. In this examination, a systematic mapping study (SMS) was conducted with a set of six research questions. A total of 1711 studies published from 2009 to 2019 were obtained. Following a filtering process, a collection of 74 primary studies were selected. As a result, the contribution of this study is declared as follows:

- Demonstrate existing threats and attacks on data privacy and solutions to serve personal data.
- Outline metrics and measures that are used to assess the current solutions for privacy in MCC.
- Illustrate the current state-of-the-art of data privacy exercises utilized in MCC and highlight the types of research and contribution areas that are used in mobile cloud computing.
- Highlight open research issues of privacy and data protection in MCC.

This article is constructed as follows: Section 2 presents background and motivation for the study. Section 3 presents the related work. Section 4 describes the research method. Section 5 presents conducting the study. Section 6 shows and discusses the results. Section 7 illustrates the key findings. Section 8 clarifies the thread to the validity. Section 9 presents the conclusion of this study.

## 2. Background and motivation

This section presents a general background of mobile cloud computing, privacy and data protection, and the needs for a systemic mapping study.

### 2.1. Mobile cloud computing

Today, mobile devices such as smartphones provide users with greater connectivity and accessibility to services and applications [1]. Even though mobile technology continues to expand, modern mobile terminals suffer limitations associated with poor computational resources, low memory size, and small disk capacity [1]. Cloud computing provides a robust approach to the delivery of services by incorporating existing computing technologies. In cloud computing, three service delivery models appear to account for most deployments: Infrastructure-as-a-service (IaaS), Software-as-a-Service (SaaS), and Platform-as-a-Service (PaaS) [5].

The concept of Mobile Cloud Computing (MCC) has emerged out of mobile technology and cloud computing [1–3]. It is an approach that aims to enable mobile terminals to access robust and reliable cloud-based computing that facilitates the optimal utilization of resources. Moreover, MCC presents opportunities for improving the portability and scalability of services [1].

### 2.2. Privacy and data protection

Several researchers have reported that privacy in the definition adopted by the organization for Economic Cooperation and Development [4] is “any information relating to a recognized or identifiable individual (data subject).” In fact, the concept of privacy is vast and has a different perspective depending on countries, cultures, or jurisdictions.

To be more precise, privacy is not just about hiding information, but it is a legitimate control over personal data since no one may get personal information without the consent of the owner unless there are laws that allow access to such information [6], for example, income information that the tax authorities can get from employers [6].

The issue of privacy in MCC is getting nowadays more attention; however, numerous existing privacy laws and regulations are needed to impose the standards for the collection, maintenance, use, and disclosure of personal information that must be satisfied even by cloud providers [7]. In addition, a number of studies reported that there is always increasing the privacy risk in hosting your data in someone else’s hands [7].

### 2.3. The need for a systematic mapping study

Currently, a considerable number of papers have been published on MCC with a growing interest in privacy and data protection. Along with this advance in MCC, our research group has found the following:

- The results of the existing studies in privacy and data protection were not highlighted.
- The ongoing trends in privacy and data protection were not determined.
- The metrics used to assess current solutions were not aggregated.
- The research type facets and the contribution type facets used in MCC were not aggregated.
- The current open research issues with future research directions were not demonstrated.

The aim of this investigation is to highlight the results of the existing studies in privacy and data protection in MCC through a systematic mapping study (SMS). The purpose of a systematic mapping study is to present an adjustable, impartial, and reliable assessment of a particular research topic [3]. Also, SMS is used to highlight the current state-of-the-art and to determine the trends of the research domain.

## 3. Related works

In recent years, a number of reviews and surveys have been published to analyze MCC in secondary studies [8–11] and are considered as related to this study. David et al. [8] focused on the various encryption techniques (and their variants) that are presently being utilized, and on possible future works that could improve privacy-oriented encryption techniques and security. Moreover, the authors tried to provide the audience with a conception about the difficulty of the algorithm being utilized in each of the studied encryption techniques. However, they did not cover other solutions or discuss current attacks and threats related to MCC.

Also, Kulkarni et al. [9] concentrated on the existing frameworks of MCC, although they did not mention other solutions. In addition, Bhatia and Verma [10] presented a state-of-the-art organization of cryptographic techniques and data security schemes in an innovative delimitation on chronological order. However, the survey only focused on threats and attacks related to the mobile cloud. Moreover, Rahimi et al. [11], investigated various security frameworks for the MCC environment, whereby most of them offload processor-heavy jobs to the cloud. The study [11] suggested some of the challenges that service providers need to address to achieve security and privacy in the MCC environment [11]. Finally, even though several reviews and surveys have been reported, two limitations remain:

- There is a need for a more systematic way of summarizing the current knowledge in MCC. It is known that the popularity of these studies is as informal literature surveys, which do not include specific research questions, search process, or defined data analysis processor data extractions.
- A few secondary studies focused on privacy and data protection in MCC, while applications based on these platforms continue to multiply.

## 4. Research method

A systematic mapping study (SMS) is a secondary study that provides a structure of the type of research papers and aggregates the results that have been declared in the domain. Also, SMS is a method for categorizing the published studies, often gives a visual summary, and map the results to highlight the current state-of-the-art and to determine the trends [12].

**Fig 1:**
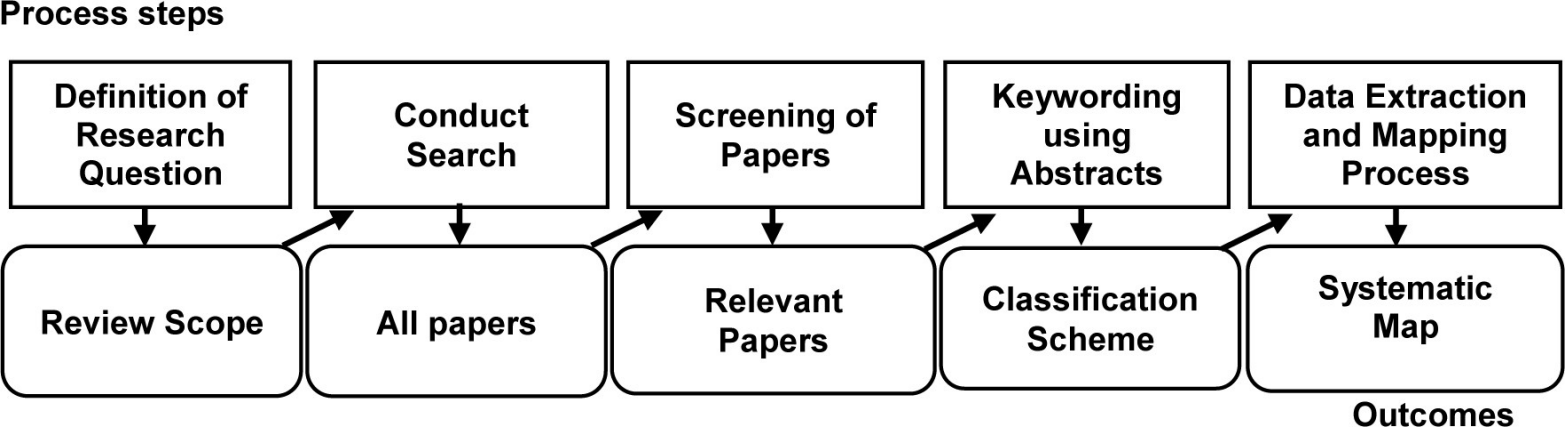
The process steps of systematic mapping [12].

In this paper, we have derived the formal guidelines of SMS from Petersen et al. [12]. As in the directive of SMS [12], SMS is performed in five steps where the outcome from each step provides the input for the next step. Fig 1 shows the SMS method, as demonstrated in Petersen et al. [12]. As shown in Fig 1, SMS is implemented as follows [12]:

**Step 1**: Define research questions and objectives to provide a general scope for the study.
**Step 2**: Define the search strategy to find the published studies from the available digital libraries.
**Step 3**: Screening process using inclusion and exclusion criteria to choose the relevant studies.
**Step 4**: Keywording to enable classification and data extraction.
**Step 5:** Data extraction and mapping process.

### 4.1. Research aim questions and objectives

The study aims to highlight the results of existing primary studies published in privacy and data protection in MCC to identify current trends and open issues in the domain. Table 1 shows our research questions and the objective of each research question.

**Table 1:** Research question and objectives.

#	Research question	Objective
RQ1	What are the current data privacy exercises in MCC?	To investigate the current data privacy exercises in MCC.
RQ2	What are the existing data privacy threats and attacks in MCC?	To identify the existing privacy threats and attacks in MCC.
RQ3	What are the privacy solutions proposed to serve personal data protection in MCC?	To identify the existing solutions that are used to preserve privacy and personal data protection in MCC.
RQ4	What are the metrics and measures that are used to assess the current solutions for privacy and data protection in MCC?	To investigate the metrics and measures that are used in assessing the current solutions for privacy in MCC.
RQ5	What research type facets and contribution type facets are used in MCC?	To classify the research type facets and the contribution type facets that are used in MCC.
RQ6	What are the current open research issues of privacy and protection in MCC?	To identify the current open research issues of privacy and protection in MCC.

### 4.2. Search strategy

As in the SMS guideline [13], the primary studies are identified by using a search string [13] derived from the research questions. An excellent way to create the search string is to structure them in terms of population, intervention, comparison, and outcome (PICO) [13]. Based on our research questions in Table 1, PICO is implemented as follows:

- **Population**: Published studies.
- **Intervention**: Privacy, data protection, mobile cloud computing, and MCC.
- **Comparison**: Not applicable.
- **Outcome**: Published studies in privacy and data protection in mobile cloud computing.

**Fig 2:**
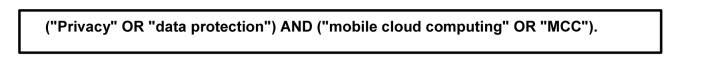
Search string.

Based on PICO, we constructed our search string as presented in Fig 2. In this SMS, the search string in Fig 2 is handled to search for studies in the available digital libraries.

### 4.3. Inclusion-exclusion criteria

Based on SMS guidelines [13], applying inclusion and exclusion criteria is crucial to filter the results [13]. Inclusion and exclusion criteria aim to obtain relevant primary studies to answer the defined research questions [13]. Table 2 illustrates our inclusion and exclusion criteria.

**Table 2:** Inclusion and exclusion criteria.

Inclusion criteria	Exclusion criteria
1) The primary study must be related to MCC and published from 2009 to 2019.	1) The primary study is written in a language other than English.
2) The primary study must present a contribution with validation or verification related to privacy or data protection in MCC.	2) The primary study is presenting a summary of a keynote, a workshop introduction, or only an abstract.
3) Peer-reviewed primary studies.	3) Studies about other issues other than privacy or data protection in MCC.
4) The primary studies that were written in the English language.	

### 4.4 Keywording and classification for data extraction

For the SMS data extraction and classification, the SMS method [14] declared the following:

- **Classification scheme:** is a process of reading the abstracts, look for keywords and concepts that reflect the contribution of the primary study [13]. The classification scheme aims to:
  ➢ Ensure that the desired results were covered in the SMS [14].
  ➢ Aid in introducing a set of categories that represent the underlying population for the study [14].
  ➢ Develop a high-level understanding of the nature and contribution of the selected primary studies [13].
- **Keywording:** is utilized to implement the classification scheme in SMS as follows:
  ➢ First, read the abstracts and searched for keywords [14].
  ➢ Second, identify the context related to the objective of the study and the scheme will be updated [14].
- **Scheme:** When having the classification scheme in place, the relevant articles are sorted into the scheme, i.e., the actual data extraction takes place [13].

**Fig 3:**
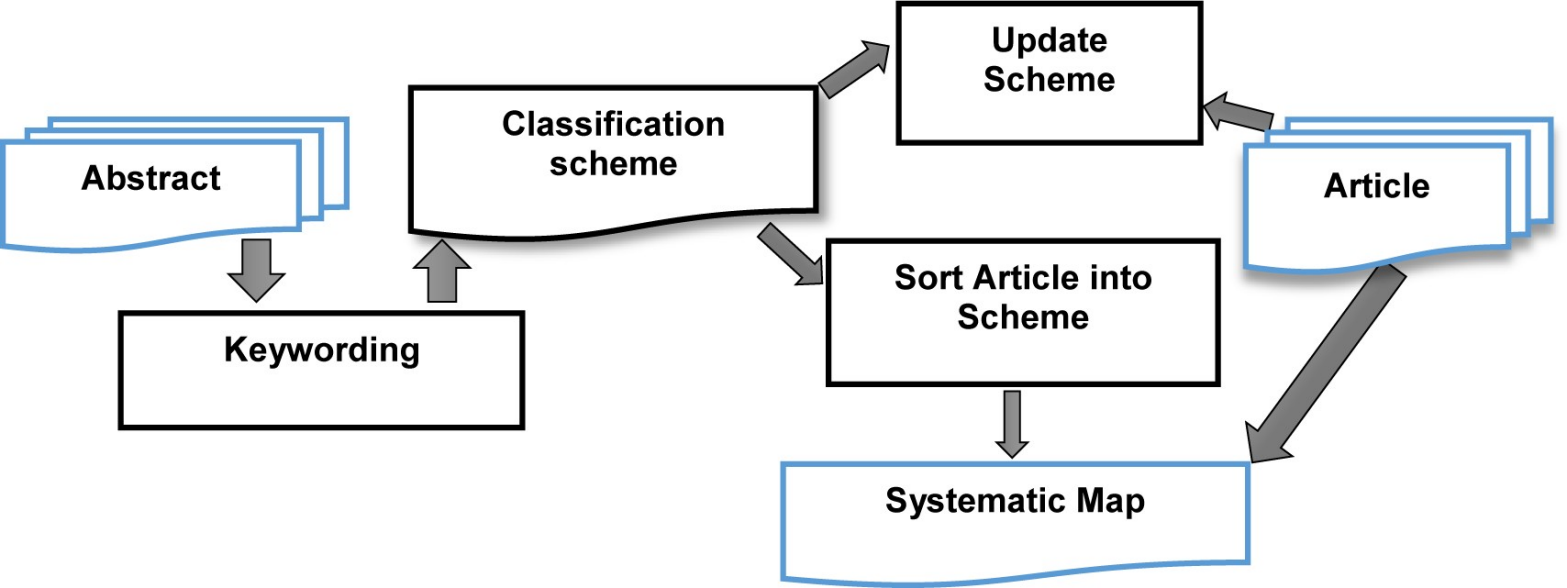
Classification scheme [14].

As presented in Fig 3, the classification scheme is implemented as follows:

1. **Keywording:** is the process of reading the abstract and searching for keywords to identify the context related to the objective of the SMS [14].
2. **Sort Article into scheme:** is the process of sorting the scheme after adding an article into scheme [14].
3. **Update scheme:** is the process of modifying the scheme after adding a primary study context to the scheme [14].

### 4.5 Data extraction and mapping process

As demonstrated in the SMS method [13], in this study, we use a data extraction form to gather the SMS data. In addition, when having the classification scheme in place, the actual data extraction of the relevant articles in this study is sorted into the scheme as follows:

- Excel tables were utilized to document the data extraction process [13].
- The frequencies of publications in each category were analyzed from the final table [13].

To investigate the trends, as in the SMS method [13], we focused on the frequencies of publications for each category to identify which categories have been emphasized in past research and thus to identify gaps and possibilities for future research. Also, different ways of presenting and analyzing the results were utilized as follows:

- The summary of the statistics is illustrated in the form of tables, showing the frequencies of publications in each category [13].
- A bubble plot is illustrated to report the frequencies [13]. Bubble plot is basically two x-y scatterplots with bubbles in category intersections. The size of a bubble is proportional to the number of articles that are in the pair of categories corresponding to the bubble coordinates [13].

## 5. Conducting SMS

In this section, we present the systematic mapping study that we have conducted using the SMS method presented in Section 4.

### 5.1. Selecting and filtering relevant studies

**Fig 4:**
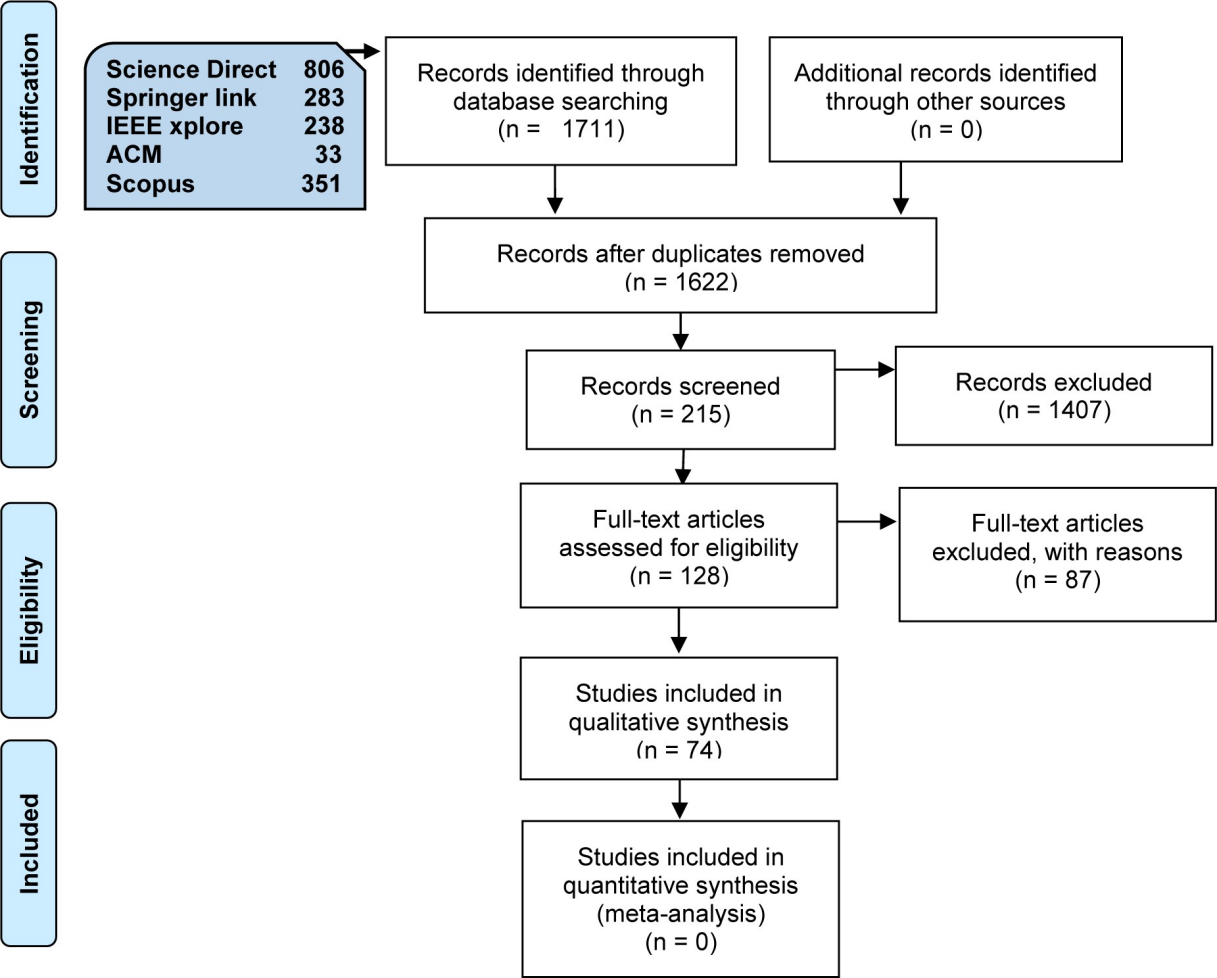
PRISMA flow diagram.

In this study, we applied PRISMA (Preferred Reporting Items for Systematic Reviews and Meta-Analyses) guidelines [15] as an evidence-based for reporting the outcome of the search results to clarify the eligible, included or excluded primary studies in this investigation. Fig 4 demonstrates the resulting articles from each database and the screened primary studies for this study using the PRISMA guideline.

As Fig 4, in this investigation, five digital databases we selected to search for relevant studies, including IEEE Xplore, Science Direct, Springer Link, ACM Digital Library, and Scopus. Then, we utilized our search string, as presented in Fig 2, to search for studies in the selected databases. As a result, 1711 studies were obtained and screened as follows:

- **By article type:** only the studies presented in conferences, magazines, and journals venues initially selected.
- **By subject:** only the studies related to privacy, data protection, mobile cloud computing, and MCC initially nominated.
- **By title:** only the studies related to mobile cloud computing initially nominated.

Finally, after screening by year, article type, subject, and title, a total of 215 studies were initially selected and presented in Table 3.

**Table 3:** The results of the search for relevant studies.

Results Database	Search result	Screen by the last ten years	Screen by Article type	Screen by subject	Screen by title
**Science Direct**	806	693	461	264	78
**Springer link**	283	231	93	93	16
**IEEE xplore**	238	232	227	200	47
**ACM**	33	23	23	23	9
**Scopus**	351	351	290	252	65
**Total**	**1711**	**1530**	**1094**	**832**	**215**

In filtering the retrieved studies, a total of 87 studies were excluded based on our inclusion and exclusion criteria (Table 2). Also, 39 duplicated studies were eliminated. In addition, we read a sum of 89 studies in a comprehensive analysis. The comprehensive analysis is a process of reading the whole primary study and decide to include or exclude it after a complete investigation on the actual contribution on exactly and only on the privacy and data protection in mobile cloud computing. Finally, a total of 74 primary studies were selected for SMS. Table 4 shows the results of filtering the retrieved studies.

**Table 4:** The results of filtering the retrieved studies.

Results Database	Search result	Comprehensive analysis			Final selection
		Remaining after inclusion and exclusion	Remaining after remove duplicated studies	Remaining after comprehensive analysis	
**Science Direct**	78	37	31	31	31
**Springer link**	16	13	7	3	3
**IEEE Xplore**	47	27	23	20	20
**ACM**	9	5	4	4	4
**Scopus**	65	46	24	16	16
**Total**	**215**	**128**	**89**	**74**	**74**

### 5.3. Analysis and classification

**Fig 5:**
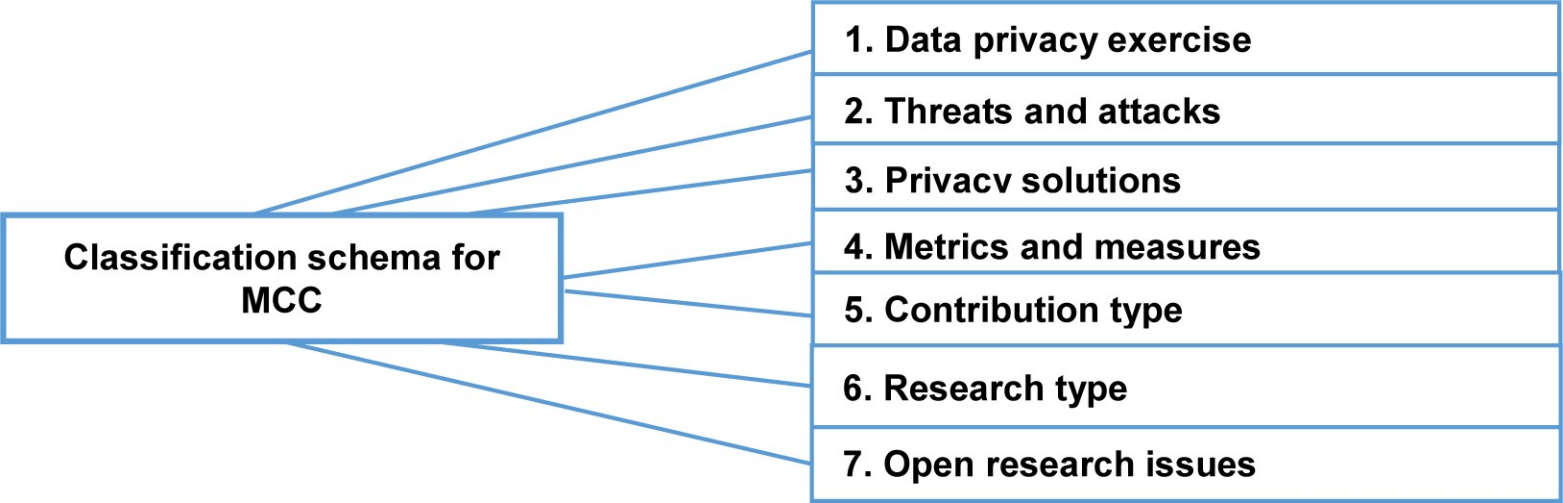
A classification scheme for a systematic mapping study in MCC.

In this study, we carried out a classification scheme through keywording as declared in Section 4.4. First, we read the abstracts of the 74 selected primary studies and searched for keywords. In addition, we read the introduction and conclusion sections of each of the selected primary studies to produce the classification scheme. As an outcome, Fig 5 shows our classification scheme.

As presented in Fig 5, seven main aspects were defined as follows:

- **Data privacy exercises:** It denotes the methods of controlling and implementing privacy solutions in mobile cloud computing [16]. Also, it concerns the demonstration of practice policies of data access using different mechanisms [17] that governed by the policies of MCC service providers, state regulations and roles.
- **Threats and attacks**:
  ➢ **Threat:** Potential for infringement of security, which exists when there is a situation, capacity, activity, or occasion that could violate security and cause harm. That is, a risk is a possible peril that may misuse a vulnerability [18].
  ➢ **Attack**: A violation of system security that derives from an intelligent threat. This intelligent work is a purposed attempt (especially in the concept of a technique or method) to avoid the security policy of a system and security services [18].
- **Privacy Solutions**: These are computational methods serving issues related to authentication, authorization, encryption, access control, and trust.
- **Metrics**: Privacy metrics are the privacy parameters that are required in measuring the level of privacy in MCC or the privacy service provided by a given solution to MCC [19].
- **Research type**: We adopted an existing classification (Wieringa, Maiden, Mead, & Rolland, 2006), which is divided into six classifications: Validation Research, Solution Proposal, Evaluation Research, Philosophical Paper, Opinion Paper, and Experience Paper [20]. **S1 Appendix of Appendix A** shows the types of research with the definitions [20] used in our mapping study.
- **Contribution type**: For the contribution type facets, we have used the categories from Petersen et al., (2008): Model, Formal Study, Method, System, and Experience [20]. **S1 Appendix of Appendix B** shows the definitions of the contribution type facets used in our mapping study.
- **Open research issues**: is a new challenge noted by the researchers in the existing studies in the area.

## 6. Results and discussion

In this section, we present and discuss the answers to the research questions of this study.

### 6.1. RQ1: What are the current data privacy exercises in MCC?

In this study, we have identified eight data privacy exercises; these eight exercises have been highlighted in the selected primary studies for implementing privacy solutions in MCC. Table 5 illustrates the identified data privacy exercises in the selected primary studies.

**Table 5:** Data privacy exercises.

#	Data privacy exercise	Primary studies
1	**Setup (system, initial, account, algorithm)**	[21, 22, 23, 24, 25, 26, 27, 28, 29, 30, 31, 32, 33, 34, 35, 36, 37, 38, 39, 40, 41, 42, 43, 44,45,46,47,48,49,50,51,52,53,54]
2	**Cryptography**	[21, 55, 24, 25, 26, 27, 28, 29, 30, 31, 36, 37, 39, 41, 56, 57, 58, 59, 44, 45, 46, 47, 60, 50, 51, 52, 53, 54, 61, 62]
3	**Authentication**	[21, 22, 63, 64, 65, 28, 29, 32, 66, 33, 67, 68, 69, 36, 35, 37, 70, 39, 41, 57, 43, 71, 72, 45, 48, 49, 51, 73]
4	**Accounts creation**	[22, 63, 30, 66, 33, 35, 70, 39, 41, 56, 42, 57, 74, 43, 71, 75, 48, 49, 73]
5	**Verification**	[21, 64, 26, 28, 29, 66, 34, 67, 37, 40, 42, 43, 71, 59]
6	**Access control**	[23, 30, 39, 74]
7	**Steganography**	[76, 55, 25]
8	**Reputation**	[77, 23, 42]

In addition, more details are necessary to understand those exercises presented in Table 5; those data privacy exercises are defined as follows:

- **Setup:** is concerning the adaptation of the initial public parameters of system, account, and algorithm for privacy and data protection in MCC [26, 27].
- **Cryptography:** is defined as the method of preserving information by using codes, such that it can only be read and interpreted by those for whom the information is targeted [18].
- **Authentication:** it denotes the assurance that the communicating entity is the one that it claims to be [18].
- **Accounts creation:** It represents the registration of a mobile device or user to a cloud server is an onetime process wherein the user information (ID, password) are Setup, and some encrypted files are exchanged [70].
- **Verification:** is utilized to illustrate the information that corroborates the binding between the entity and the identifier [18].
- **Access control:** is the prevention of unauthorized use of a resource [18].
- **Steganography:** is used for hiding plaintext messages by concealing the existence of the message [18].
- **Reputation:** is one of the components of trustworthiness measures. The reputation establishes based on the recommendations from the MCC users [78].

**Fig 6:**
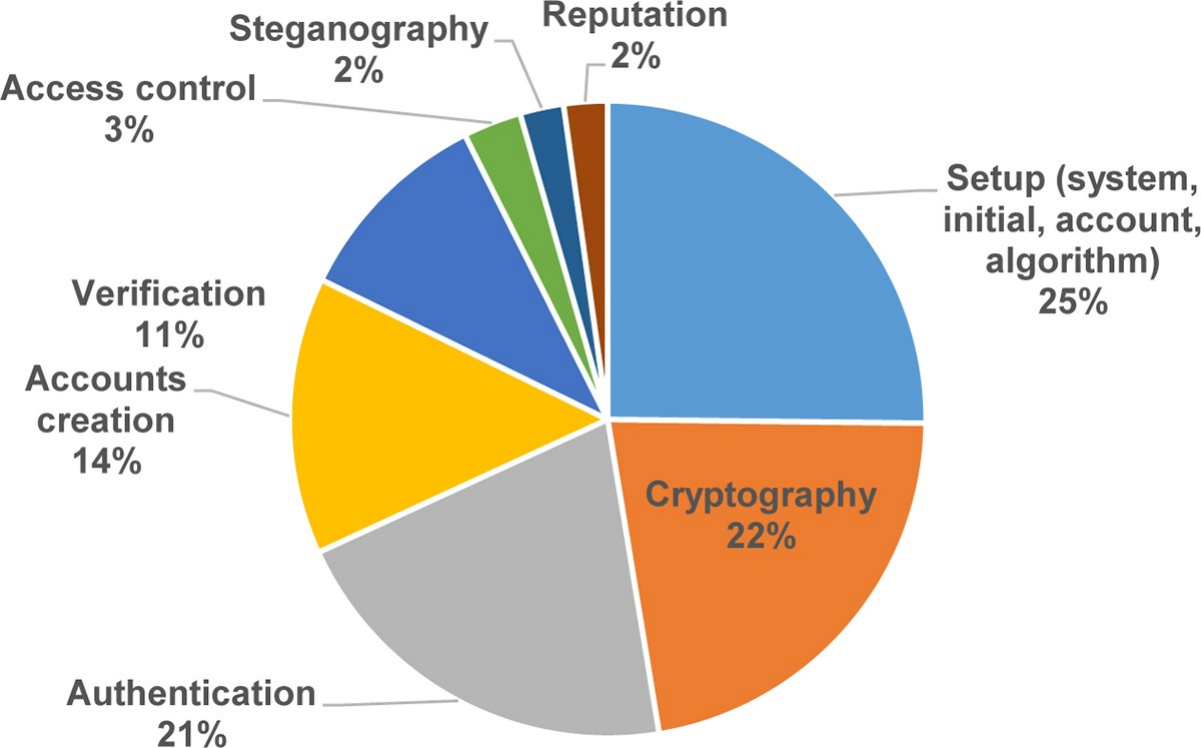
Data privacy exercises.

Fig 6 shows the percentage of studies related to data privacy exercises based on the number of studies. As presented in Fig 6, the results show that the selected primary studies focused on setup, cryptography, authentication, account creation, and verification in 25%, 22%, 21%, 14%, and 11% of studies, respectively. On the other hand, access control, steganography, and reputation have scored the lowest percentage with less than 5% each.

**Fig 7:**
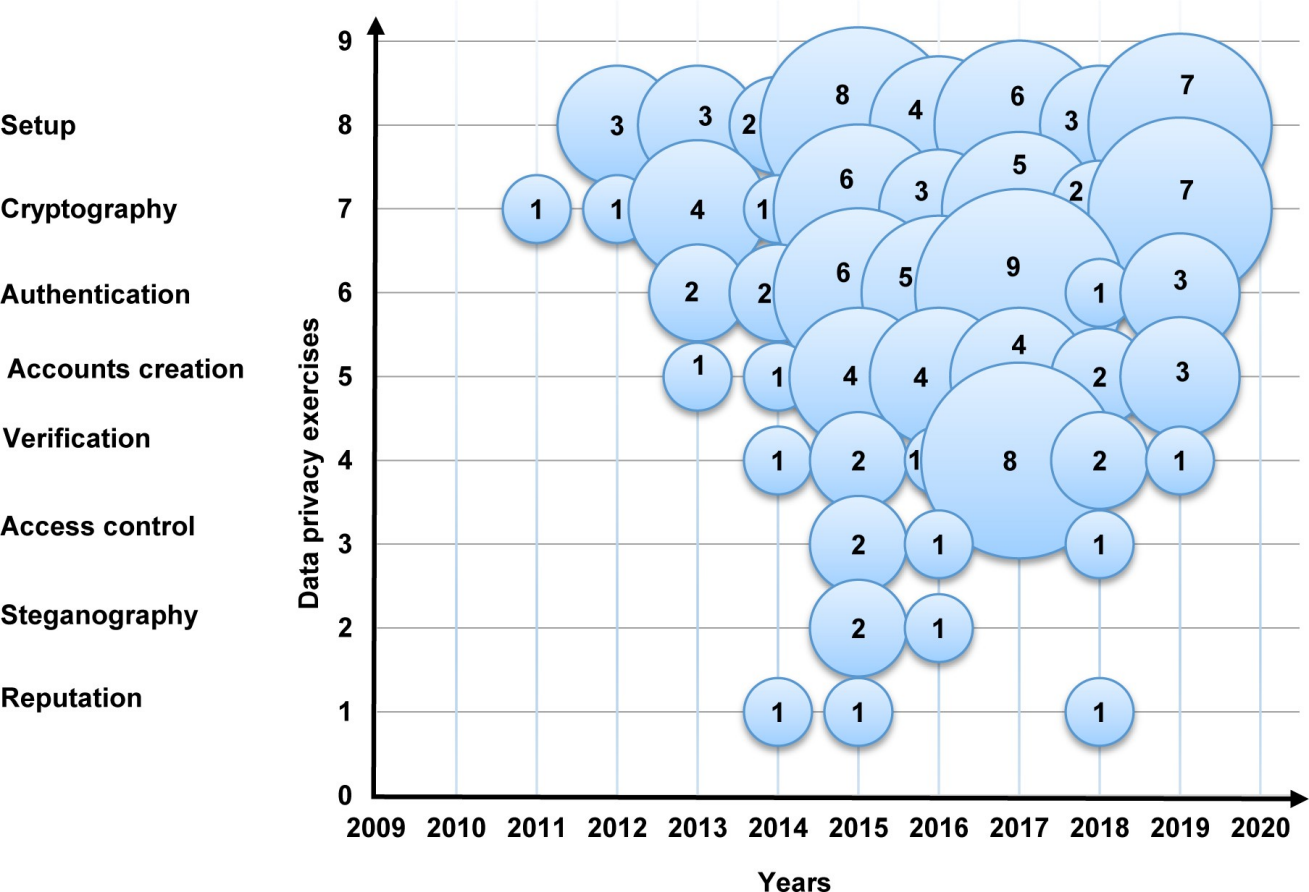
Bubble plot of the data privacy exercises.

Moreover, Fig 7 is a bubble plot of data privacy exercises in the selected primary studies; the X-axis represents the years, and the Y-axis represents the data privacy exercises. As illustrated in Fig 7, the number of research rises towards the setup, cryptography, authentication, and accounts creation. Conversely, the number of research decreased towards verification, access control, steganography, and reputation.

### 6.2. RQ 2: What are the existing data privacy threats and attacks in MCC?

In this investigation, we have identified 17 data privacy threats and attacks in MCC. Table 6 shows the identified threats and attacks in the selected primary studies.

**Table 6:** Threats and attacks.

#	Threats and attacks	Primary studies
1	**Unauthorized includes users, persons, and access**	[76, 55, 22, 77, 23, 24, 25, 63, 27, 65, 28, 32, 34, 67, 69, 36, 79, 38, 70, 40, 41, 56, 42, 57, 74, 58, 75, 44, 80, 50, 51, 81, 82, 83]
2	**Data privacy**	[76, 55, 23, 25, 26, 27, 64, 30, 31, 34, 84, 36, 35, 7, 40, 85, 86, 42, 59, 44, 46, 60, 49, 87, 51, 81, 88, 53, 89]
3	**Leakage of user privacy**	[27, 32, 66, 67, 84, 37, 90, 91, 85, 86, 41, 42, 43, 92, 58, 72, 59, 44, 47, 80, 49, 51, 73, 93]
4	**Data misuse**	[55, 63, 26, 27, 65, 31, 66, 67, 37, 79, 38, 40, 86, 56, 42, 74, 43, 71, 82, 54, 61]
5	**Untrusted service provider**	[21, 76, 55, 77, 24, 25, 63, 26, 27, 28, 66, 67, 36, 70, 40, 58, 72, 87, 52, 83, 62]
6	**Disclosing information or data**	[21, 55, 77, 23, 66, 67, 91, 86, 41, 56, 58]
7	**Man-in-the-middle attacks**	[64, 65, 32, 84, 70, 41, 42, 74, 82]
8	**Impersonation attacks**	[63, 27, 33, 41, 57, 43, 75, 48]
10	**Phishing attacks**	[27, 64, 66, 44, 45, 47, 82, 94]
9	**Identity theft**	[64, 70, 42, 47, 48]
11	**Collusion attacks**	[23, 27, 29, 47, 50]
12	**Eavesdropping attacks**	[26, 64, 67, 93]
13	**Internal attacks**	[77, 23, 67]
14	**Improper security policies and practices in some locations**	[55, 27, 61]
15	**Internal multi-layer attacks**	[77]
16	**Inference attack on user privacy**	[63]
17	**Data breach threats**	[27]

**Fig 8:**
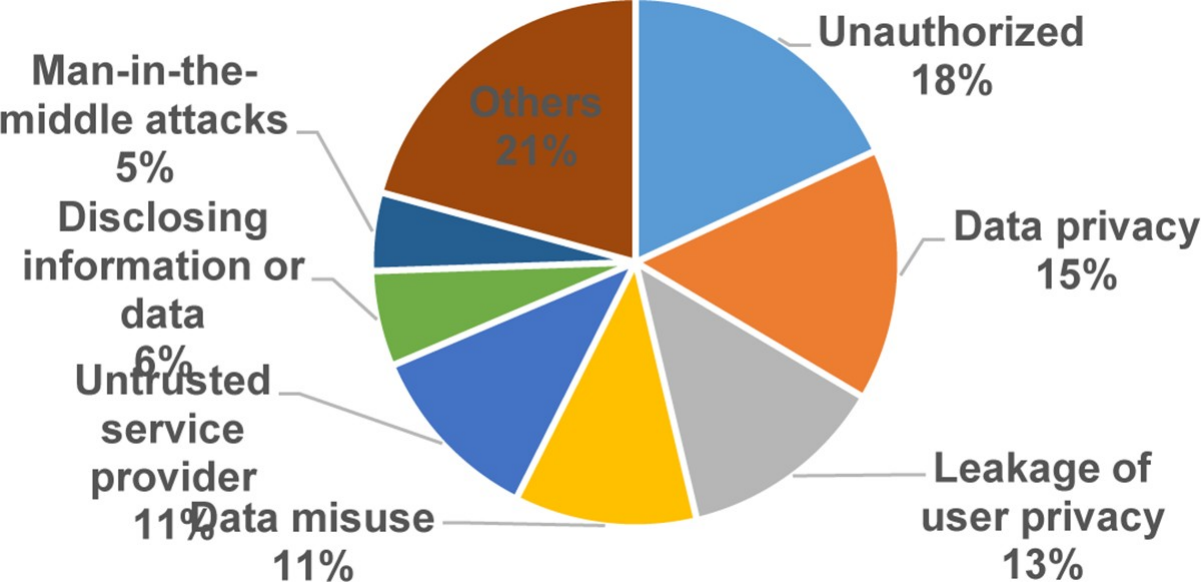
Threats and attacks.

Fig 8 displays the percentage of primary studies related to threats and attacks based on the number of studies. As demonstrated in Fig 8, the most common threats and attacks are unauthorized threats and attacks including users, persons, and access with 18% (34), data privacy with 15% (29), leakage of user privacy 13% (24), data misuse (21) and untrusted service provider (21) represented 11% each. On the other hand, disclosing information or data (11) represented 6%, man-in-the-middle attacks (9) represented 5%, and the rest of the threats got 21%, respectively.

**Fig 9:**
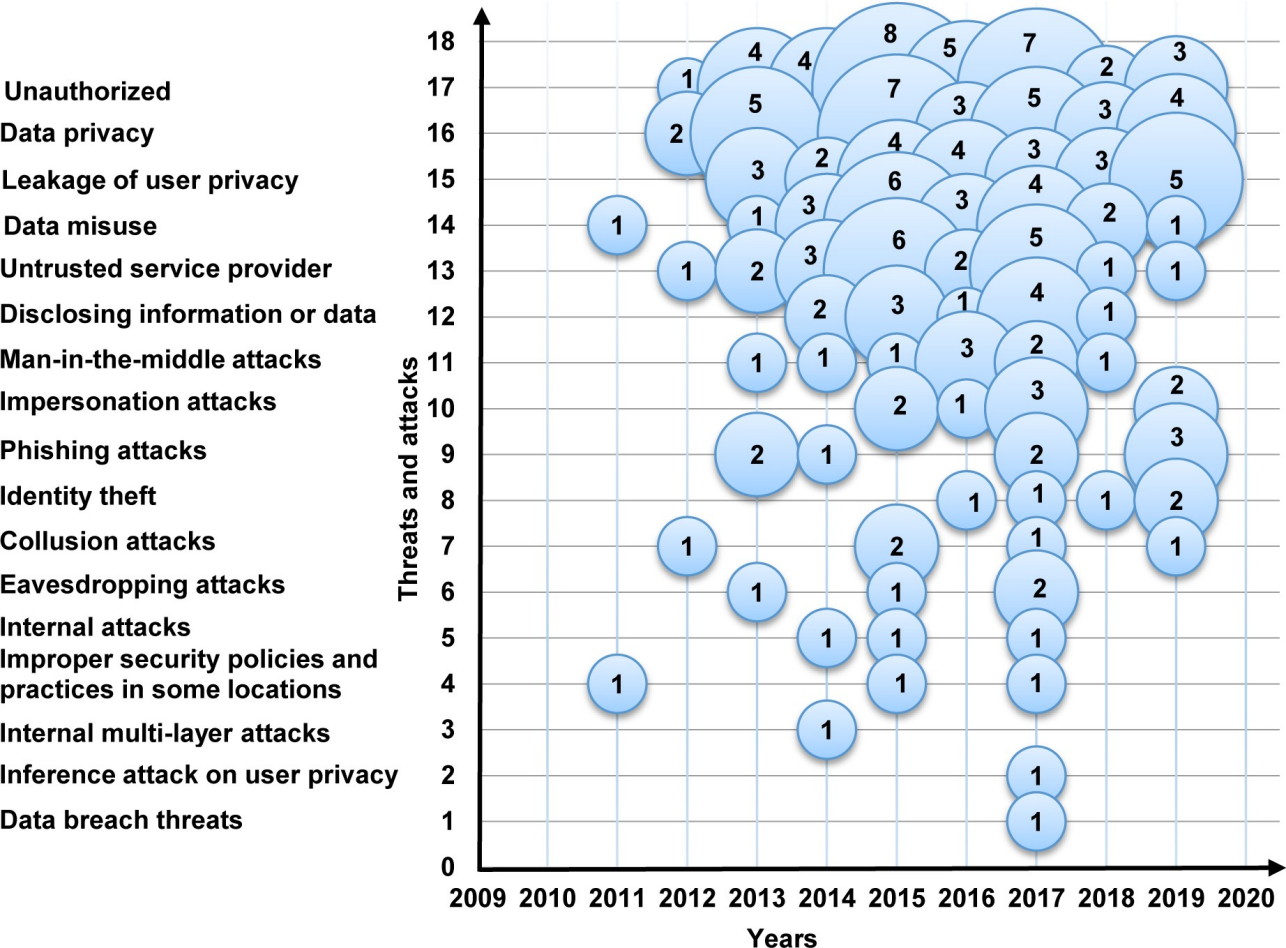
Bubble plot of the threats and attacks.

Furthermore, Fig 9 is a bubble plot of threats and attacks, the X-axis represents the years, and the Y-axis represents the threats and attacks. The results show that unauthorized, data privacy, leakage of user privacy, and phishing attacks are relatively dominant in the field. In contrast, eavesdropping attacks, internal attacks, improper security policies and practices in some locations, internal multi-layer attacks, inference attacks on user privacy, and data breach threats are losing momentum.

### 6.3. RQ3: What are the privacy solutions proposed to serve personal data protection in MCC?

As shown in Table 7, four solutions used to preserve the privacy in MCC in the selected primary studies. The solutions include encryption, authentication, access control, and trust.

**Table 7:** Privacy solutions.

#	Solutions	Primary study
1	**Encryption**	[21, 76, 55, 25, 26, 65, 28, 29, 30, 31, 66, 34, 7, 79, 90, 40, 91, 42, 74, 59, 44, 45, 46, 47, 60, 87, 50, 51, 81, 52, 53, 93, 83, 54, 61]
2	**Authentication**	[22, 63, 64, 32, 33, 67, 68, 69, 35, 70, 41, 57, 43, 71, 72, 75, 48, 49, 73, 94]
3	**Access control**	[23, 24, 27, 84, 36, 37, 39, 85, 86, 56, 58, 80, 62]
4	**Trust**	[77, 89]

**Fig 10:**
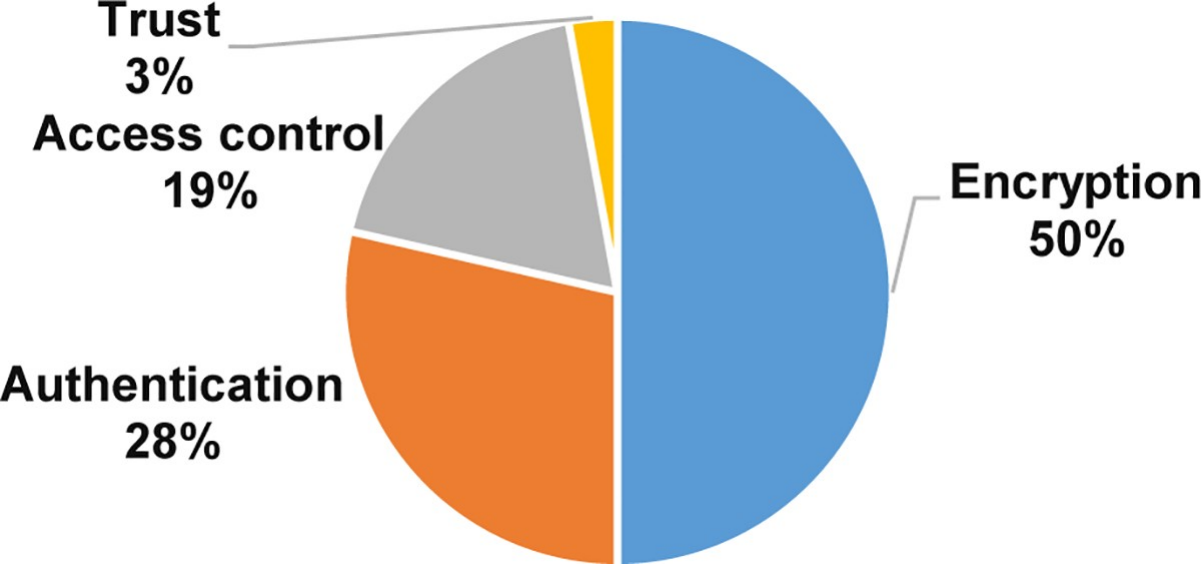
Privacy solutions.

Fig 10 displays the percentage of studies related to privacy solutions based on the number of studies. The outcome shows that the research focused on encryption, authentication, and access control solutions in 50%, 28%, and 19% of studies, respectively. We observed that researchers have started to propose trust as a solution in this domain since we found two studies presented the trust solutions.

**Fig 11:**
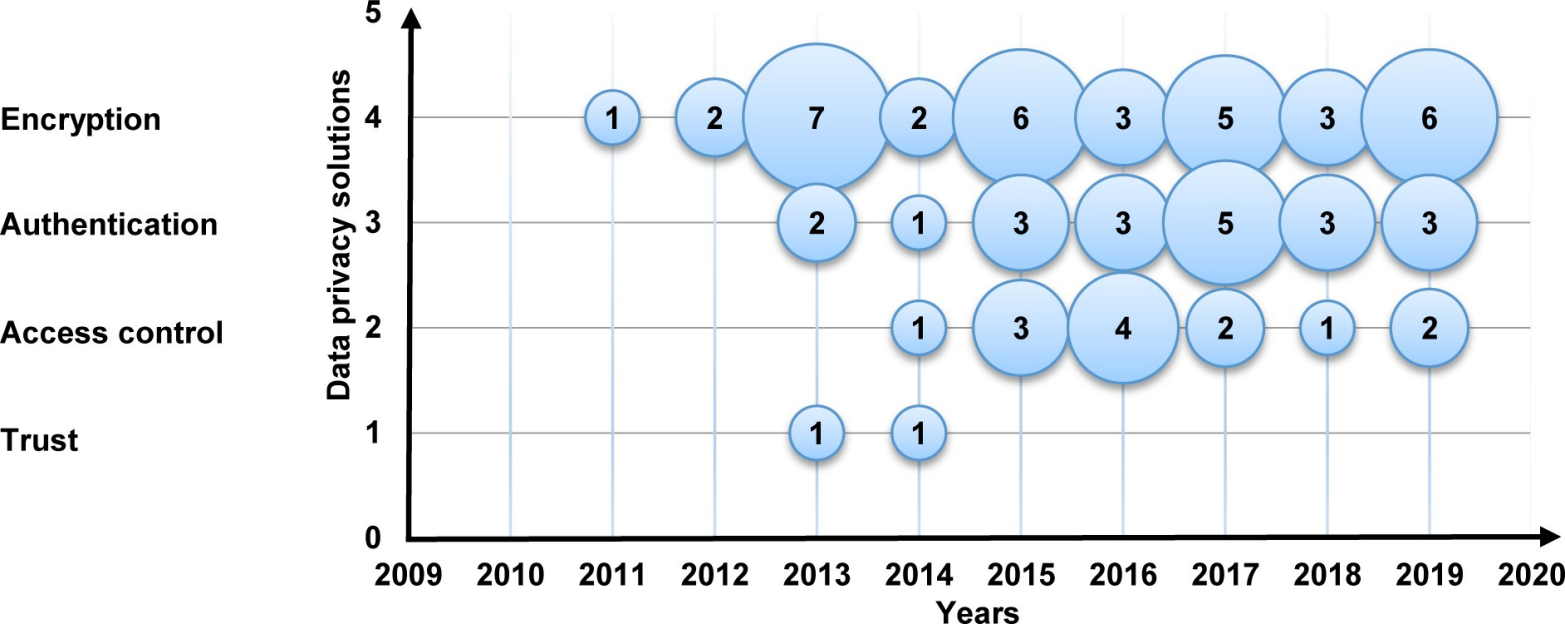
Bubble plot of privacy solutions.

Moreover, Fig 11 is a bubble plot of privacy solutions with the X-axis representing the years and the Y-axis representing data privacy solutions. The result in Fig 11 determines that the amount of research is increasing towards the encryption and the authentication data privacy solutions. On the other hand, research into trust data privacy solutions is abating.

### 6.4. RQ4: What are the metrics and measures that are used to assess the current solutions of privacy and data protection in MCC?

As shown in Table 8, we divided the answer into two parts as follows:

- The first part of Table 8 presents the resources usage metrics, where we found that the highest utilized metric is time consumption, which is represented in 32 studies, followed by communication overhead in 26 studies. The results display that energy consumption, memory consumption on mobile devices, and turnaround-time resources usage metrics received the least attention in the selected primary studies.
- The second part of Table 8 shows the contained solution robustness metrics. The results show two studies for each of the effective recommendation rate, accuracy, authentication request, and authentication response. Also, the results show one study for each of the data randomization, a malicious node detection and management performance (MDP), the addition of new users, operations required, authorities, and privacy and reliability factors.

**Table 8:** Metrics and measures.

Types	Metrics and measures	Primary studies
Resources Usage metrics	**Time consumption**	[21, 55, 22, 24, 63, 26, 27, 29, 31, 66, 33, 34, 67, 68, 37, 7, 38, 39, 40, 41, 42, 57, 71, 58, 59, 75, 44, 47, 48, 49, 51, 88]
	**Communication overhead**	[24, 64, 65, 32, 66, 33, 67, 68, 36, 7, 91, 85, 86, 41, 56, 42, 57, 71, 58, 75, 49, 50, 52, 53, 93, 83]
	**Energy consumption**	[21, 25, 65, 7, 38, 41, 45, 46, 60, 53, 93]
	**Memory consumption on mobile**	[21, 55, 36, 7]
	**Turnaround time**	[21, 46]
Solution robustness metrics	**Effective recommendation rate**	[77, 23]
	**Accuracy**	[23, 31]
	**Authentication request**	[70, 73]
	**Authentication response**	[70, 73]
	**Data randomisation**	[55]
	**Malicious node detection and management performance (MDP)**	[77]
	**Addition of new user**	[24]
	**Operations required**	[63]
	**Authorities**	[29]
	**Privacy and reliability factors**	[38]

**Fig 12:**
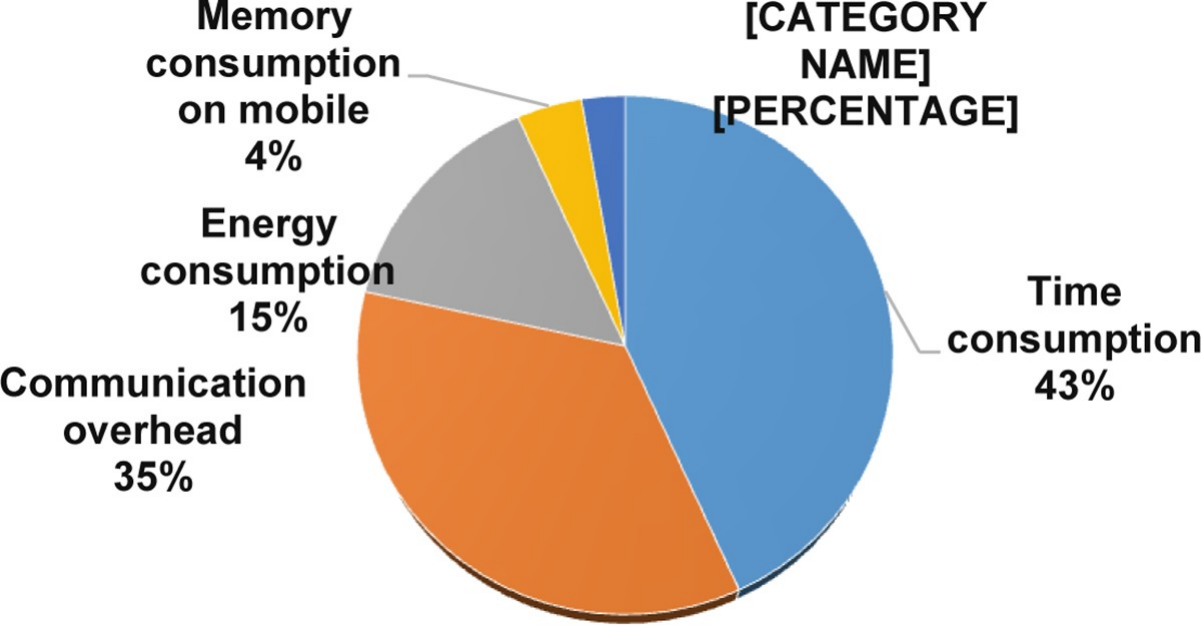
Resources usage metrics.

As illustrated in Fig 12, the time consumption is the most used metric resulted in 43%. Followed by communication overhead metrics with 35%. Finally, energy consumption, memory consumption, and turnaround time are presented in 15%, 4%, and 3%, respectively.

**Fig 13:**
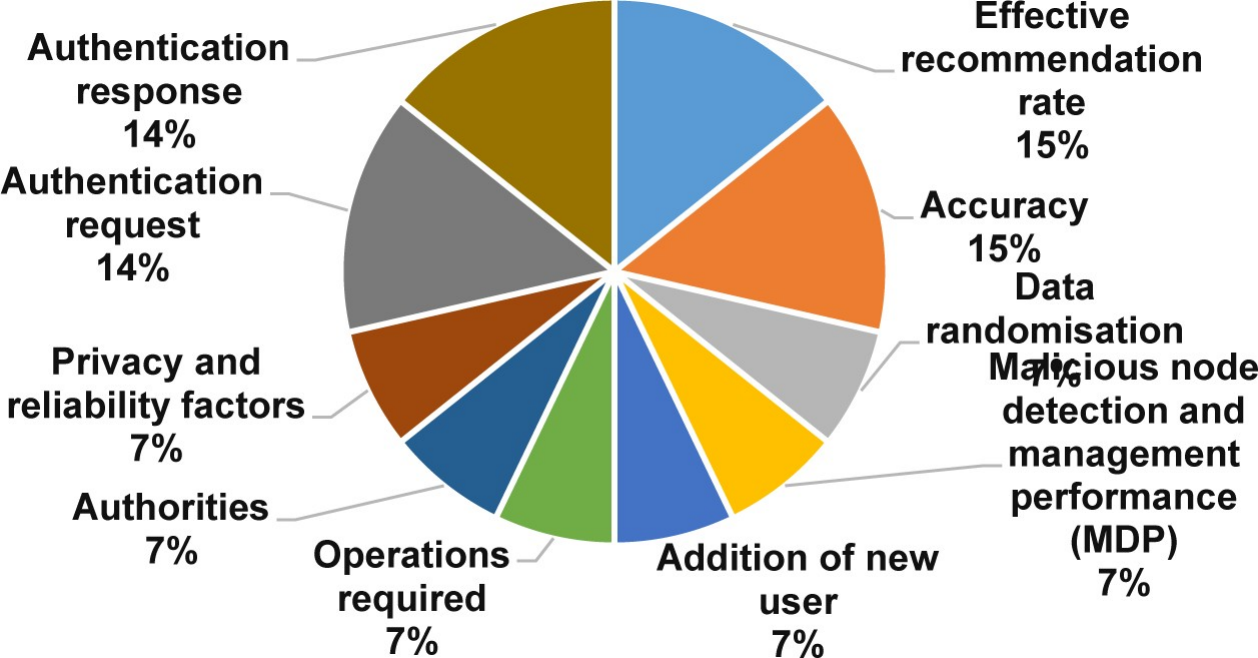
Solution robustness metrics.

As expounded in Fig 13, the effective recommendation rate, accuracy, authentication response, and authentication request are the most used metrics with 15%, 15%, 14%, and 14%, respectively. One the other hand, the result shows that most of the solution robustness metrics were employed in less than 8% of the selected primary studies.

**Fig 14:**
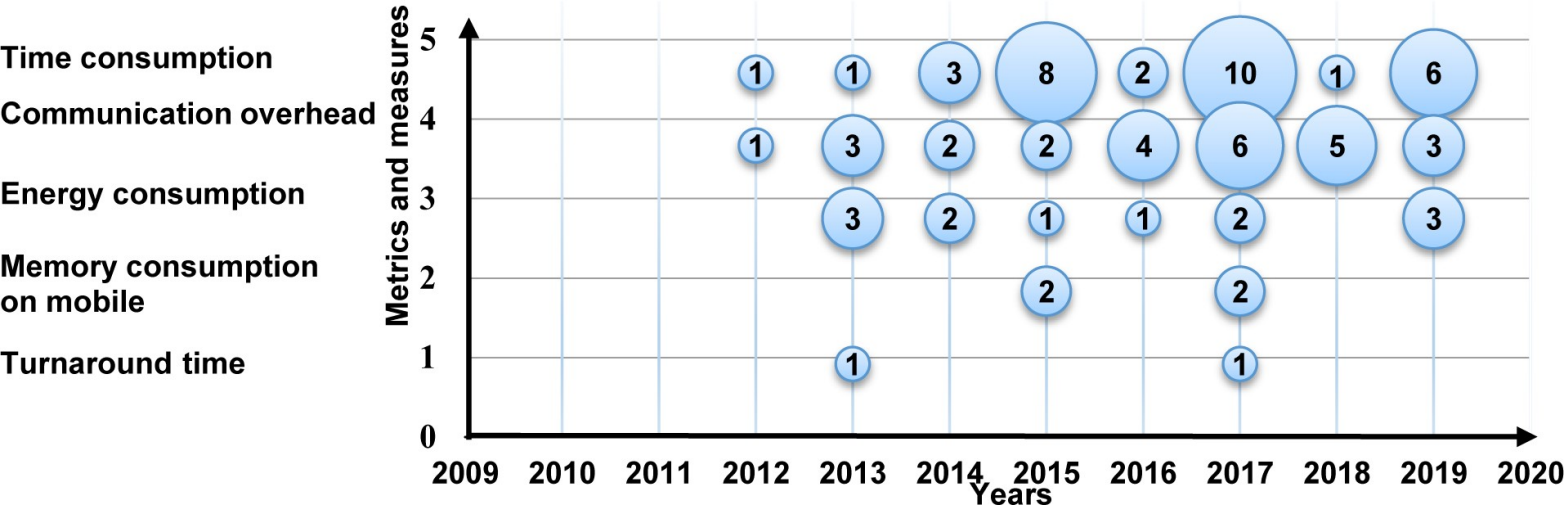
Bubble plot of the metrics and measures.

For recognizing metrics and measures trends in MCC, we present the trends in a bubble plot in Fig 14, the X-axis represents the years, and the Y-axis represents metrics and measures. The outcome indicates that the amount of research in the selected primary studies is increasing towards time consumption, overhead communication, and energy consumption metrics. On the other hand, the number of studies in memory consumption and turnaround time is receiving less attention.

### 6.5. RQ 5: What research type facets and contribution type facets are used in MCC?

**Fig 15:**
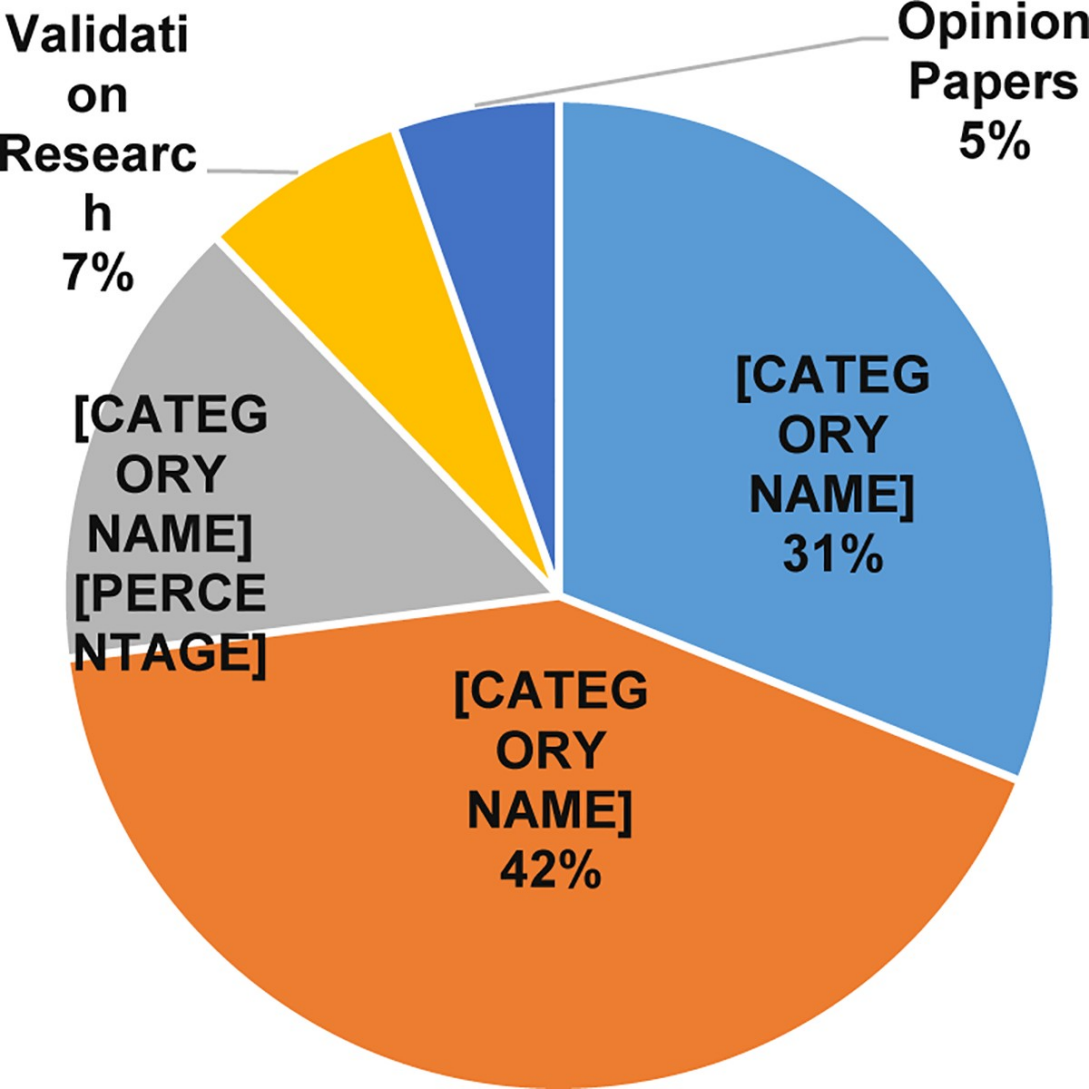
Research type facets for MCC.

To answer the first part of this question, we studied the proportion of papers by research type, as shown in Table 9 and Fig 15. Our studies found the solution proposals are the most published studies with 31 papers (42%), followed by the evaluation research with 23 papers (31%). In contrast, there are 11 philosophical papers (14%), five validation research (6%), and four opinion papers (5%).

**Table 9:** Research type facets and contribution type facets.

#	Research type facets	Contribution type facets	Primary study
1	**Solution Proposal**	Model	[21, 77, 24, 36, 90, 72, 60]
		Method	[76, 55, 25, 28, 31, 68, 37, 92, 71, 59, 75, 47, 49, 87]
		System	[63, 26, 29, 39, 61]
		Formal Study	[79, 57, 46, 53, 54]
2	**Evaluation Research**	Model	[64, 30, 34, 38, 85, 56, 58, 44, 88, 83]
		Method	[32, 69, 70, 41, 43, 81]
		System	[40, 45, 80]
		Formal Study	[86, 73, 89, 93]
3	**Validation Research**	Model	[65, 66, 7, 48, 62]
4	**Philosophical Papers**	Model	[22, 33, 67, 35, 91, 42, 74, 50, 51, 52]
		Method	[27]
5	**Opinion Papers**	Model	[23]
		Method	[84]
		System	[82, 94]

**Fig 16:**
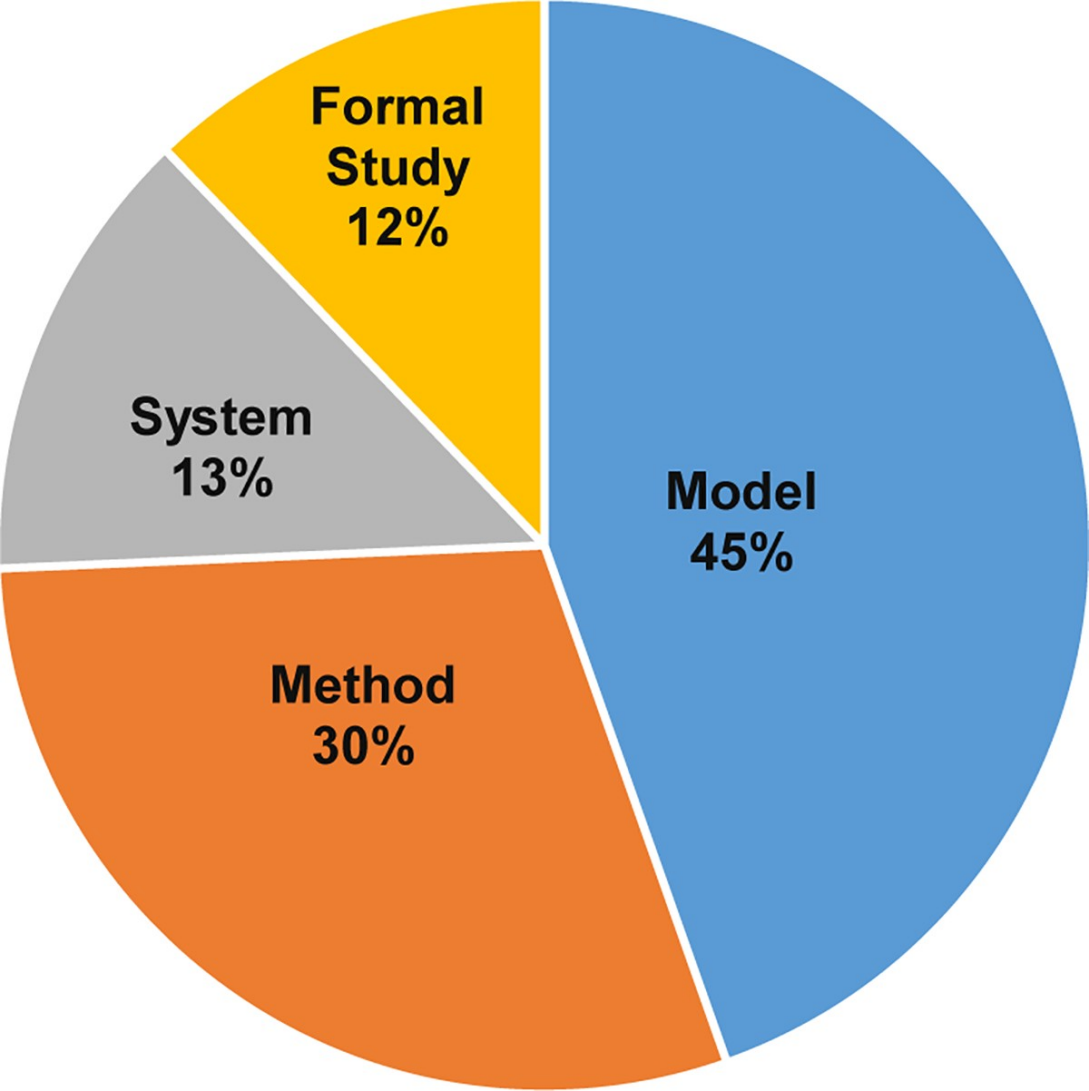
Contribution type facets for MCC.

To answer the second part of the question, we studied the proportion of papers by research type, as shown in Table 9 and Fig 16. Our studies found the most popular contribution type is the model with 33 papers (45%), followed by the method with 22 papers (30%). In contrast, there are only ten system contributions (13%), and nine Formal studies (12%).

**Fig 17:**
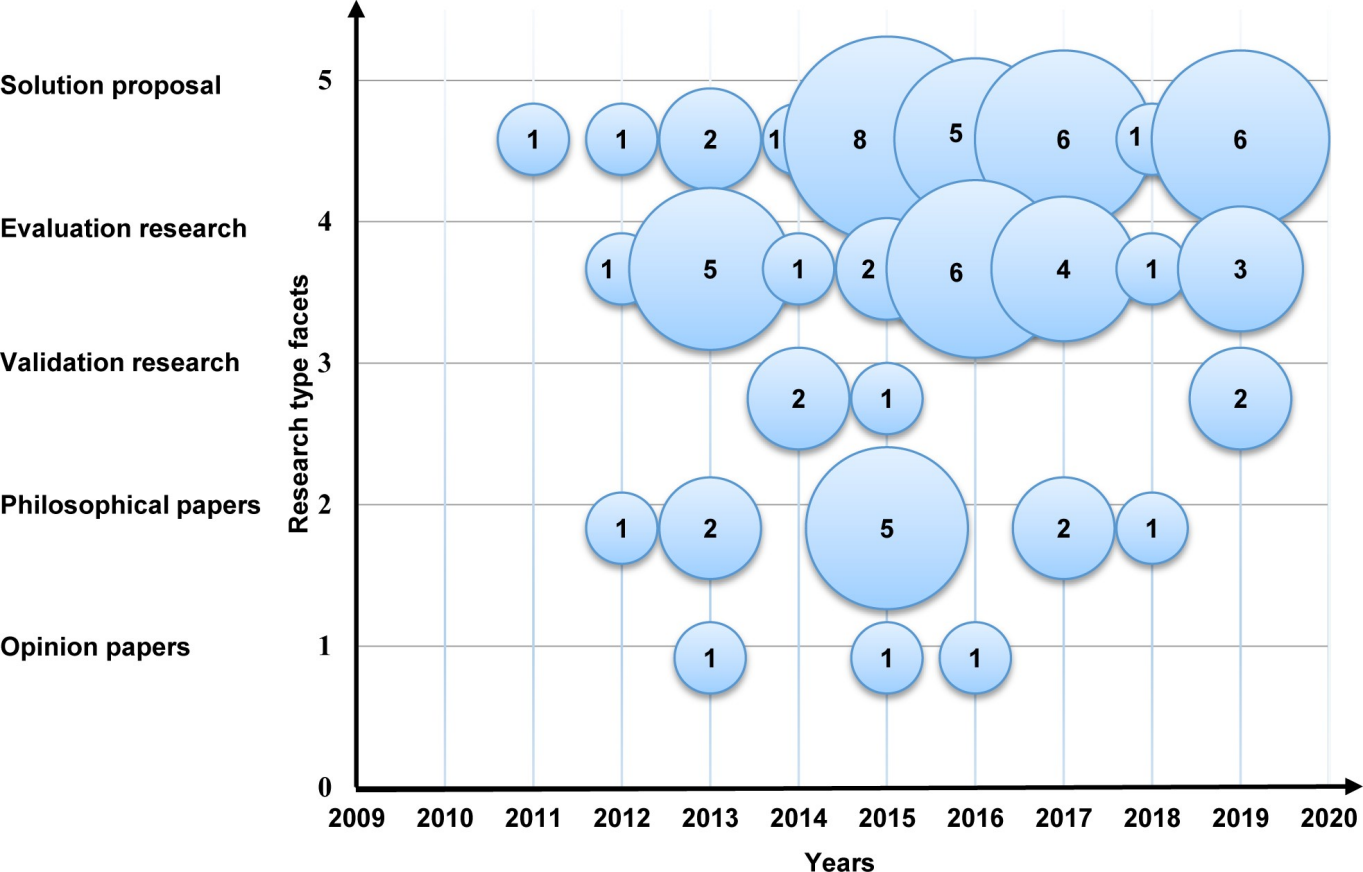
Bubble plot of the research type facets.

To discover the research type facets in MCC trends, we illustrate the trends in a bubble plot (Fig 17), the X-axis represents the years, and the Y-axis represents the research type. As demonstrated in Fig 17, the amount of research in the selected primary studies is increasing towards the solution proposal and the evaluation research. On the other hand, the number of validation research and opinion paper research type facets are decreasing.

**Fig 18:**
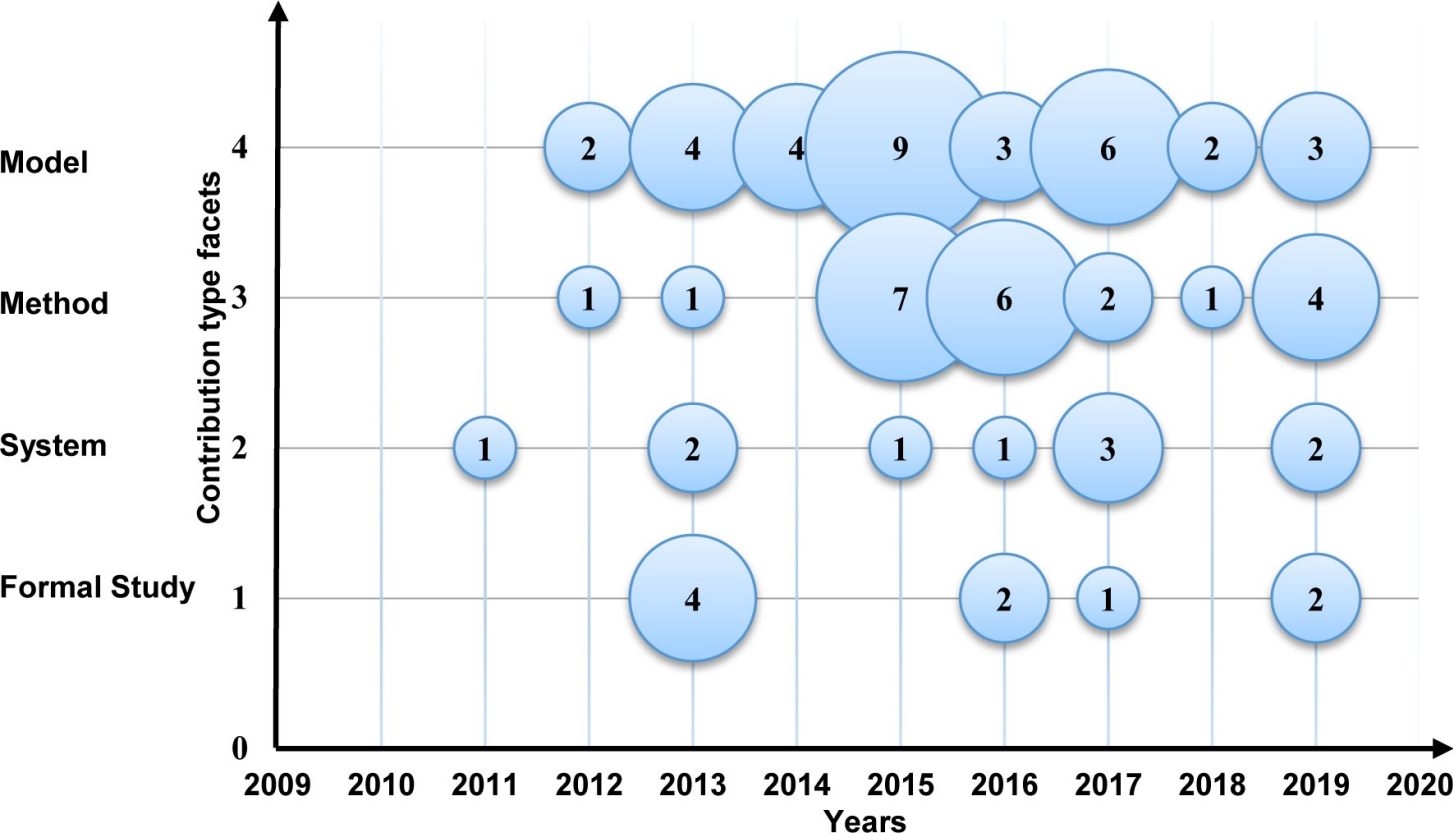
Bubble plot of the contribution type facets.

To discover the contribution type facets in MCC, we illustrate the trends in a bubble plot (Fig 18), the X-axis represents the years, and the Y-axis represents the contribution type. The outcome shows that the models and the methods are relatively dominant in the field, and the systems and the formal studies are losing momentum in the domain.

### 6.6. RQ 6: What are the currently open research issues of privacy and data protection in MCC?

In this study, we have identified nine main open research issues with 23 examples of future research directions suggested by the authors in privacy and data protection in MCC. Table 10 shows the identified open research issues in the selected primary studies.

**Table 10:** Open research issues.

Open issue	Examples of future research directions suggested by the authors
**Security [23, 7, 71, 44, 45, 61]**	Secure access control model for big data applications in MCC to implement more secure and fine-grained access control [23].
	Cloud Security System for secure communication over the cloud [7].
	Against such risks as loss or unauthorized access, destruction, use, modification, or disclosure of data [7].
	The problem of data redundancy in the cloud when the user registration again [71].
	Evaluation of having a single static sink in mobile multiple sinks [45].
	Exploring machine learning approaches to enhancing the level of data secrecy [44].
	Investigate security of monitoring, auditing, and misuse detection in the mobile cloud system [61].
**Authentication [65, 42, 43]**	Inadequate dynamic federation and agile mechanisms in current IDM systems [65].
	Analyze the annotations and semantic-based relationship identification for the attributes in policy trees [42].
	Design a secure and efficient authentication scheme for distributed mobile cloud services [43].
**Privacy [23, 83, 61]**	Enhance the power of the adversary, to protect the individual privacy against an active adversary [83].
	Combine the encryption or signature based privacy preserving technology with the secure access control model to improve the performance of the privacy preserving [23].
	Investigate the application scenarios that require data sharing between cloud private domain and public domain [61].
**Encryption [21, 79, 69]**	Re‐encryption scheme with no disclosure of the user information to any entity involved in the system [21].
	Hash algorithm to be applied in mobile applications to distributed systems [79].
	Construct more expressive (fully secure) anonymous ABE schemes with fast decryption [69].
**Energy consumption [47, 60]**	Investigation of the increase in the lifetime of a mobile battery by reducing the computational complexity involved in encryption and decryption algorithms [47].
	Evaluation of energy efficiency versus security issue at cost and energy efficiency of offloading [60].
**Trust [24, 49]**	Implementation of inter-relation between different trusted leaders like data owner and cloud to protect data and owner privacy without revealing the owner's content and identity to the cloud [24].
	Evaluation of efficient token revocation and reduce the communication round for the Internet of Things.[49]
**Architectures [91]**	Peer-to-peer architectures without a dedicated entity to serve as SAS [91].
**V2arious attacks [37]**	Resist various attacks in mobile cloud systems to enhance privacy protection for location-based services [37].
**Testing [79]**	Measure the resistance of the mobile cloud server in serving users [79].

**Fig 19:**
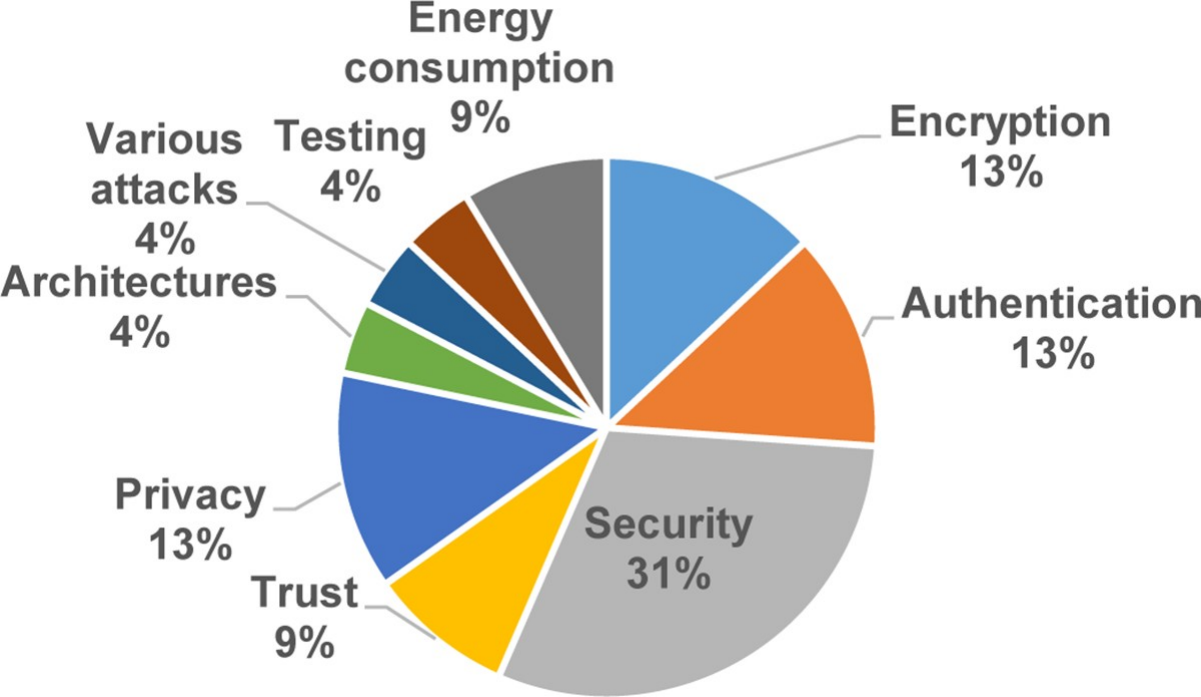
Open research issues in privacy and data protection.

Furthermore, Fig 19 displays the open research issues in privacy and data protection based on the number of studies. As illustrated in Fig 19, security, authentication, privacy, and encryption were getting momentum in 31%, 13%, 13%, and 13%, respectively. On the other hand, energy consumption, trust, various attacks, architectures, and testing addressed in less than 10% of the selected primary studies for each of them.

## 7. Key findings

In this study, a systematic mapping study was conducted with a set of six research questions. A total of 1711 studies published from 2009 to 2019 were obtained. Following a filtering process, a set of 74 primary studies were selected. In this section, we summaries the key findings of this study as follows:

- **The current data privacy exercise in MCC**: This study shows that the exercises of cryptography, authentication, account creation, and verification were getting significant attention in 93% of the selected primary studies. In contrast, access control, steganography, and reputation with less attention in less than 8% of the selected primary studies. Also, our results show that the amount of research is increasing in the setup, cryptography, authentication, and accounts creation. Conversely, the outcome shows that the research in verification, access control, steganography, and reputation are losing momentum.
- **The data privacy threats and attacks in MCC**: The results of this SMS show that the issues of unauthorized, data privacy, leakage of user privacy, data misuse, and untrusted service provider were receiving the most consideration in 68% of the selected primary studies. On the other hand, internal attacks, improper security policies and practices in some locations, internal multi-layer attacks, inference attacks on user privacy, and data breach threats were received less consideration with fewer than 6% of the selected primary studies. Also, our results show that unauthorized, data privacy, leakage of user privacy, and phishing attacks are relatively dominant. Conversely, the outcome indicates that the research in eavesdropping attacks, internal attacks, improper security policies and practices in some locations, internal multi-layer attacks, inference attacks on user privacy, and data breach threats have the lowest studies in the domain.
- **The privacy solutions proposed to serve personal data protection in MCC**: The results of this SMS show that the encryption, authentication, and access control of the solutions in MCC were getting the highest attention in 97% of the selected primary studies. Trust solutions had the lowest concern in the field with less than 4%. Furthermore, the amount of research is increasing in encryption and the authentication of data privacy solutions in MCC. Contrary to expectations, the outcome shows that the research in the trust solutions in MCC is less likely than expected with only 3% of the selected primary studies.
- **The metrics and measures that are used to assess the current solutions of privacy and data protection in MCC**:
  ➢ It is interesting to note that this study identified five resources usage metrics and ten solution robustness metrics. In resource usage metrics, around 78% of primary studies assess the time consumption and the communication overhead. In solution robustness metrics, an effective recommendation rate and accuracy were gotten 30% of primary studies.
  ➢ In resource usage metrics, the amount of research is increasing in time consumption and communication overhead metrics and measures. In contrast, energy consumption, memory consumption, and turnaround time are utilized in less than 23% of the selected papers. Furthermore, less than 4% of the primary studies used turnaround time metrics, which indicated that the turnaround time measures are less popular in the domain.
  ➢ In solution robustness metrics, the recognized data randomization, a malicious node detection and the management performance, the addition of new users, operations required, authorities, privacy and reliability factors, authentication requests, and authentication, were gotten less than 8% of the selected papers. On the other hand, the amount of research is increasing in accuracy and effective recommendation of metrics and measures in MCC. Conversely, the outcome shows that the research on privacy and reliability is not dominant in the area.
- **The research type facets in MCC:** The results show that the solution proposals and evaluation research got considerable attention in 73% of the selected primary studies. The validation research and the opinion papers with the lowest examinations with less than 13% of the selected primary studies. The amount of research is increasing in the solution proposal and evaluation research type. Conversely, the outcome shows that the research in the validation research and opinion paper is losing momentum.
- **The Contribution type facets in MCC:** The results of this SMS show that the models and the methods got the highest attention in 75% of the selected primary studies. Also, systems and formal studies had gotten the lowest studies in the field with less than 26% of the selected primary studies. In addition, our results show that the amount of research is increasing in the models and the methods of the contribution type facets. Surprisingly, the research in the systems and formal studies are decreased in the selected primary studies.
- **Open research issues:** In this study, we identified the new challenges in privacy and data protection in MCC, which were noted by the researchers in the selected primary studies. As presented in the previous SMS [95], the issues that emerged ten years ago are still considered open issues [95]. Our exploration shows that there are open research issues in encryption, authentication, security, trust, signature-based privacy, architectures, various attacks, testing, and energy consumption. In this SMS, as illustrated in Table 10, 23 examples of future research directions suggested by the authors are useful for research activities in the future.

## 8. Threats to validity

The process of SMS is not infallible as with any secondary research method. There are many risks to consider for ensuring the validity of this SMS study. In this part, we describe and relieve the risks to the validity of this study to mitigate the potential risks. The risks include the search criteria, digital databases, and inclusion and exclusion criteria [96].

### 8.1. Search criteria

In this examination, the highest attention paid for choosing the most useful search strings. In particular, the construction of the search string is a threat to the validity of this study [96]. To mitigate this threat, our search string is derived based on PICO criteria [13]. PICO criteria are popular and widely used in the SMS, and this would enable us to retrieve the wanted studies in the search result and mitigate the threat.

### 8.2. Digital databases

For this study, the selection of databases, including IEEE Xplore, Science Direct, Springer Link, ACM Digital Library, and Scopus is a threat to the validity of the study since related studies would not be included in those databases. To mitigate this threat, as presented in Kitchenham et al. [97], and pointed out by Dyba et al. [98], the selection of IEEE, ACM, and any two databases are enough to save time and effort for general rather than searching multiple publishers’ digital databases [97, 98]. Accordingly, in this examination, we selected five databases, including IEEE and ACM, which will mitigate the threat.

### 8.3. Inclusion and exclusion criteria

In this exploration, the rules and conditions of our inclusion and exclusion criteria are defined to be ranged with the scope of the study. The criteria stemmed from discussions within the research team. However, producing rules to recognize the initial literature to review; means that there is a threat that relevant research may be ignored if it employs various terms to that of the criteria. However, primary search terms of the study’s, namely Privacy, data protection in mobile cloud computing (MCC), are traditional, well-defined and accepted terms, which should decrease the number of ignored studies. Moreover, as the study is focused on identifying the main research in privacy and data protection in the mobile cloud computing, there is not as much of a concern with capturing research that is loosely related to the domain.

## 9. Conclusion

Mobile cloud computing (MCC) is a significant area of research emerging out of mobile devices and cloud computing [3]. In recent years, a significant number of studies have been published with a growing interest in privacy and data protection. Along with this advance in MCC, however, no specific research identified the current trends and open issues in privacy and data protection in MCC. This study highlighted current trends and open issues in privacy and data protection in MCC using the results of existing primary studies published from 2009 to 2019.

In this study, a systematic mapping study (SMS) was conducted with a set of six research questions. A total of 1711 studies published from 2009 to 2019 were obtained. Following a filtering process, a set of 74 primary studies were selected. As a result, the existing threats and attacks on data privacy and solutions to serve personal data were demonstrated. Also, the metrics and measures that are used to assess the current solutions for privacy in mobile cloud computing were aggregated. In addition, the current state-of-the-art of data privacy exercises used in the domain was identified. Moreover, the research type’s facets and the contribution type facets that are used in MCC were highlighted. Furthermore, the open research issues of privacy and data protection in MCC were demonstrated.

This result of this study shows that, for the current data privacy exercise in MCC, the number of investigations is increasing regarding the setup, cryptography, authentication, and accounts creation of data privacy exercise. Also, for data privacy threats and attacks in MCC, the results of this study show the need for research in eavesdropping attacks, internal attacks, improper security policies and practices in some locations, internal multi-layer attacks, inference attacks on user privacy, and data breach threats. In addition, our exploration shows that there are open research issues in encryption, authentication, security, trust, privacy, architectures, various attacks, energy consumption, and testing. Overall, this SMS highlighted the current state-of-the-art, and demonstrated open research issues which in turn allows us to understand the required research into privacy and data protection in MCC.

Finally, this study provides for researchers and practitioners the current state of research in the privacy and data protection in MCC, to help in implementing privacy and data protection in their applications or their investigations. In future work, we plan to conduct a survey to assess possible solutions for preserving privacy and protection in MCC.

## Supporting information

S1 Appendix The contribution type facets definitions for Systematic Mapping Study (SMS) [20]. (DOCX)

S1 Checklist PRISMA 2009 checklist (Adapted for KIN 4400). (PDF)

S1 Fig PRISMA 20009 flow diagram. (PDF)
