# Supplementary material for: Privacy and data protection in mobile cloud computing: A systematic mapping study
Source: PLoS One. 2020 Jun 11;15(6):e0234312. doi: 10.1371/journal.pone.0234312 (PMC7289432; doi:10.1371/journal.pone.0234312)
Supplement: S1 Appendix — (DOCX) [file pone.0234312.s001.docx]

**APPENDIX A**

**The contribution type facets definitions for Systematic Mapping Study (SMS) [84].**

| Research Type | **Evaluation**  **Research** | This research type provides the implemented solution and investigates a practical problem. It utilizes a sound research method (mathematical proof/logic reasoning, survey, case study, field experiment, or field study) to validate the knowledge claim. The paper contains enough discussion to provide visions for the lessons learned and the related work [84]. |
| --- | --- | --- |
|  | **Solution**  **Proposal** | This research type offers a solution technique and argues for its significance, without a whole validation. The technique must be either novel, or at least a relevant improvement of an existing technique. A proof-of-concept can be provided by means of a sound argument, a small example, or by some other means [84]. |
|  | **Validation**  **Research** | This research type differs from evaluation research in that the techniques investigated have not yet been implemented in practice and is novel. The investigation utilizes a methodologically sound and thorough research setup. Probable research methods include experiments, mathematical, mathematical analysis, prototyping, proof of properties, and simulation [84]. |
|  | **Philosophical**  **Paper** | This research type is philosophical in nature, which presents a new way of considering existing items by structuring the area in the form of a conceptual framework or a taxonomy [84]. |
|  | **Opinion Paper** | This research type contains the author’s opinion about what is good or wrong about something, or how we should do something [84]. |
|  | **Experience**  **Paper** | This research type contains case studies of one or more projects or reports on personal experiences that have been conducted, and provides lessons learned. This addressed experience must be original and relevant to practitioners [84]. |

**APPENDIX B**

**The contribution type facets definitions for Systematic Mapping Study (SMS) [84].**

| Contribution  Type | **Model** | The type of contribution is a framework or a conceptual model, system design, or software architecture [84]. |
| --- | --- | --- |
|  | **Formal Study** | The contribution type is a formal analysis, theory, or measurement of some aspect of CC, such as reliability, failure rates, and performance. The outcome may be a formalism or metric [84]. |
|  | **Method** | The contribution type is a new or extensive method, strategy, approach, algorithm, process, procedure, language, or technique [84]. |
|  | **System** | The type of contribution is the platform, environment, simulation implementation of a novel software system, or software tool [84]. |
|  | **Experience** | The contribution type is lessons learned and a description of personal experience. It is closely associated with experience papers [84]. |
