# Supplementary material for: Privacy and data protection in mobile cloud computing: A systematic mapping study
Source: PLoS One. 2020 Jun 11;15(6):e0234312. doi: 10.1371/journal.pone.0234312 (PMC7289432; doi:10.1371/journal.pone.0234312)
Supplement: S1 Fig — (PDF) [file pone.0234312.s003.pdf]

PRISMA 2009 Flow Diagram

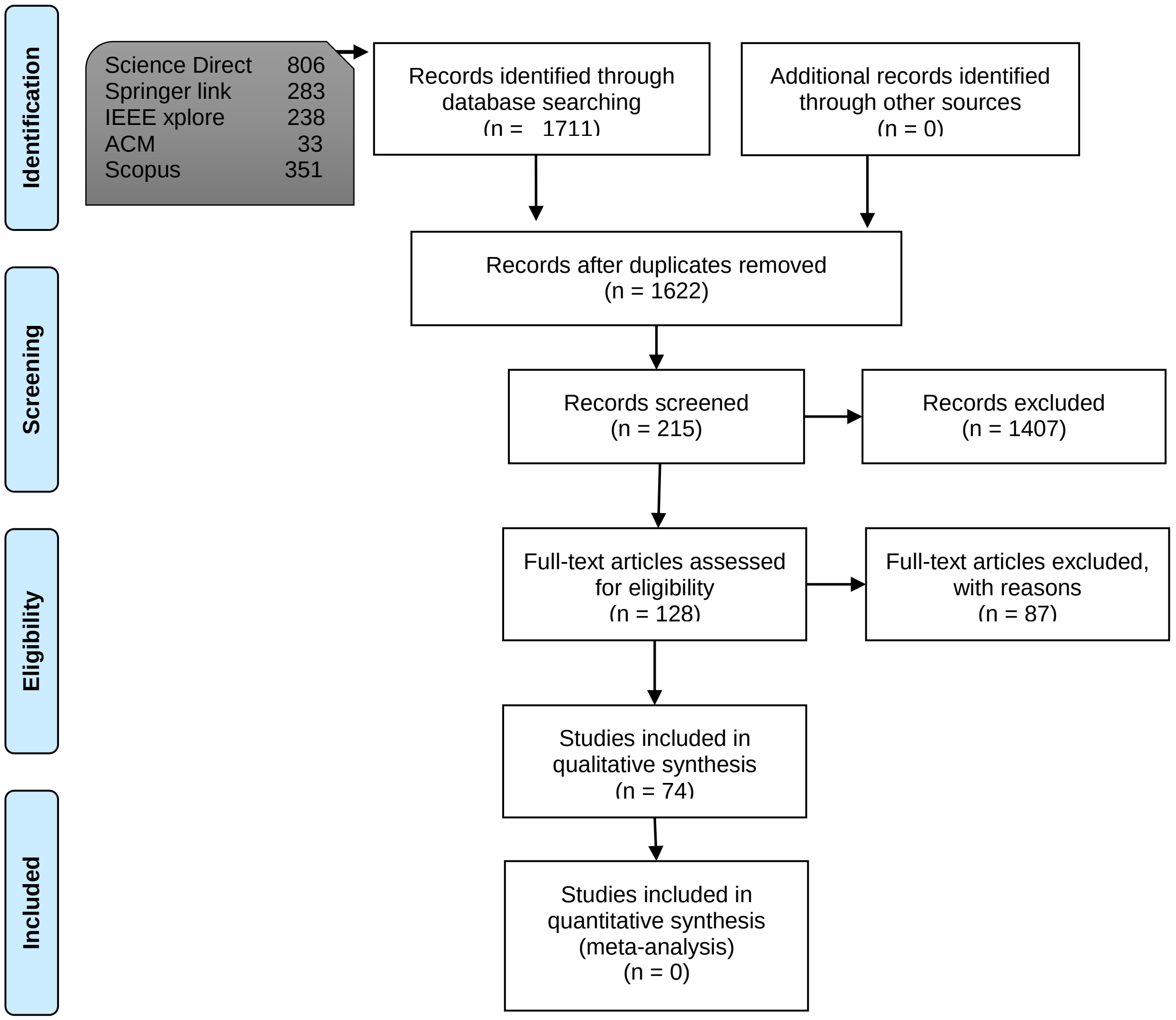

From: Moher D, Liberati A, Tetzlaff J, Altman DG, The PRISMA Group (2009). *P*referred *R*eporting *I*tems for *S*ystematic Reviews and *M*eta-*A*nalyses: The PRISMA Statement. PLoS Med 6(7): e1000097. doi:10.1371/journal.pmed1000097

For more information, visit www.prisma-statement.org.
